# Morpho-Physiological Adaptations of Rice Cultivars Under Heavy Metal Stress: A Systematic Review and Meta-Analysis

**DOI:** 10.3390/life15020189

**Published:** 2025-01-27

**Authors:** Esmeth C. Espinola, Monica Maricris N. Cabreros, Mark Christian Felipe R. Redillas

**Affiliations:** 1 Science Education Department, Br. Andrew Gonzales FSC College of Education, De La Salle University, 2401 Taft Ave., Manila 0922, Philippines; esmeth_espinola@dlsu.edu.ph; 2 Department of Biology, College of Science, De La Salle University, 2401 Taft Ave., Manila 0922, Philippines; maricris_cabreros@dlsu.edu.ph

**Keywords:** abiotic stress, heavy metals, morphology, morpho-physiology, *Oryza sativa*, physiology, rice

## Abstract

Soil contamination, including in rice fields, arises from a variety of natural processes and anthropogenic activities, leading to an accumulation of heavy metals. While extensive research has addressed the bioaccumulation of heavy metals in rice, only limited systematic reviews have examined their specific impact on the morpho-physiological traits of rice plants. This review aims to provide a comprehensive synthesis of current studies detailing the rice cultivars, types of heavy metals investigated, study designs, sampling locations, and experimental sites while systematically analyzing the morphological and physiological responses of rice cultivars to heavy metal stress. Studies show that morphological traits generally exhibit a decline under heavy metal exposure. Physiologically, rice cultivars tend to show decreased total chlorophyll and carotenoid levels, along with increased levels of malondialdehyde (MDA), hydrogen peroxide (H₂O₂), and antioxidant enzymes such as superoxide dismutase (SOD), peroxidase (POD), catalase (CAT), ascorbate peroxidase (APX), and proline. These findings suggest that plant genotype, type of heavy metal, and intensity of stress significantly modulate the morphological and physiological responses of rice, highlighting critical areas for further research in heavy metal stress tolerance in rice cultivars.

## 1. Introduction

In recent decades, rapid economic and industrial development has led to an increasing risk of heavy metal contamination in the environment. Heavy metal contaminates the soil through a wide range of routes brought about by natural processes such as soil erosion, geological weathering, and volcanic activity and anthropogenic activities such as mining, agriculture, and leaching of metals from different sources such as landfills and waste dumps [1,2]. Heavy metals possess high densities and atomic numbers compared to other metals. They cannot be broken down and are non-biodegradable, resulting in prolonged periods of concentration in the environment [3]. Heavy metals that are commonly found in contaminated soils are Chromium (Cr), Lead (Pb), Copper (Cu), Iron (Fe), Cadmium (Cd), Manganese (Mn), Nickel (Ni), Zinc (Zn), Mercury (Hg), Cobalt (Co), and Arsenic (As) [3,4,5]. However, some heavy metals, including manganese, nickel, copper, cobalt, iron, chromium, and zinc, are necessary for living organisms and play critical roles in various metabolic processes, while others are non-essential and toxic for living organisms, including arsenic, cadmium, lead, and mercury [6,7].

Plants can absorb heavy metals through phytoremediation, a process that involves root uptake, translocation, bioaccumulation, and the degradation of contaminants [5]. While plants require trace amounts of certain metals for metabolic processes, excessive heavy metal accumulation is detrimental to plant health. Similar to other environmental stresses—such as salinity, which hinders seedling growth; drought, which alters biochemical composition; and temperature extremes, which reduce productivity—heavy metal toxicity adversely impacts plant growth and yield [8,9].

Various studies on heavy metal toxicity in plants have been conducted, with particular attention to crops due to health risks associated with human consumption. Copper toxicity negatively affects the morphological traits in flax (*Linum usitatissimum* L.) by reducing plant height, technical stem length, and root length [10]. Another study reported adverse physiological and physicochemical effects of chromium in corn (*Zea mays* L.) by altering its biomass, yield, chlorophyll, and fat level contents [11]. Lead causes changes in the respiratory activities of leaves, decreases nutrition uptake through roots, alters the permeability of cell membranes and chloroplastic ultrastructure, generates reactive oxygen species (ROS), and activates certain enzymes and non-enzymatic antioxidants [12]. Excess iron ions can harm the plasma membrane and the root cells, resulting in oxidative stress and elevated reactive oxygen species generation [13]. Toxic levels of cadmium reduce grain yield, photosynthesis, mineral nutrition, growth, and seed germination [14]. In rice, it also results in genotoxicity and oxidative stress. Soybean plants exposed to excess manganese showed reductions in the stomatal conductance and CO₂ assimilation rate, which in turn led to a drop in shoot biomass [15]. In terms of nickel toxicity, aside from chlorosis and necrosis, nickel toxicity also inhibits several physiological functions in plants, such as transpiration, photosynthesis, and oxidative damage [16]. Exposure to arsenic typically causes the generation of reactive oxygen species, which can result in the synthesis of several enzymes involved in antioxidant defense as well as antioxidant metabolites [17].

Among staple crops, rice (*Oryza sativa* L.) holds economic importance, feeding more than 50% of the global population [10,18]. Asia dominates global rice production, contributing 89.7% of the total output, followed by Africa (4.9%), the Americas (4.8%), and Europe and Oceania. The leading producers include China, India, and Southeast Asian nations such as the Philippines [8,19]. Although significant research has explored the bioaccumulation of heavy metals in rice, systematic reviews on their impacts on rice’s morphological and physiological traits remain limited, often relying on older datasets. To address this gap, the present study systematically reviews recent data (2014–2024) to evaluate the morpho-physiological responses of rice cultivars to heavy metal stress, providing updated insights into this critical area of research.

## 2. Materials and Methods

### 2.1. Search Strategy

The software Publish or Perish (v. 8.12.4612.8838) [20] was downloaded from Harzing.com (accessed on 2 September, 2024) and utilized to generate lists of journal articles focusing on the morpho-physiological changes in rice plants under heavy metal stress. Multiple meta-search engines, including Crossref, Semantic Scholar, PubMed, OpenAlex, and Google Scholar, were employed to ensure comprehensive coverage of the literature. The search was restricted to publications from 2014 to the first quarter of 2024. Keywords and descriptors used during the search process included “rice cultivars”, “heavy metal”, and “morpho-physiology”.

### 2.2. Selection Criteria

**Figure 1:**
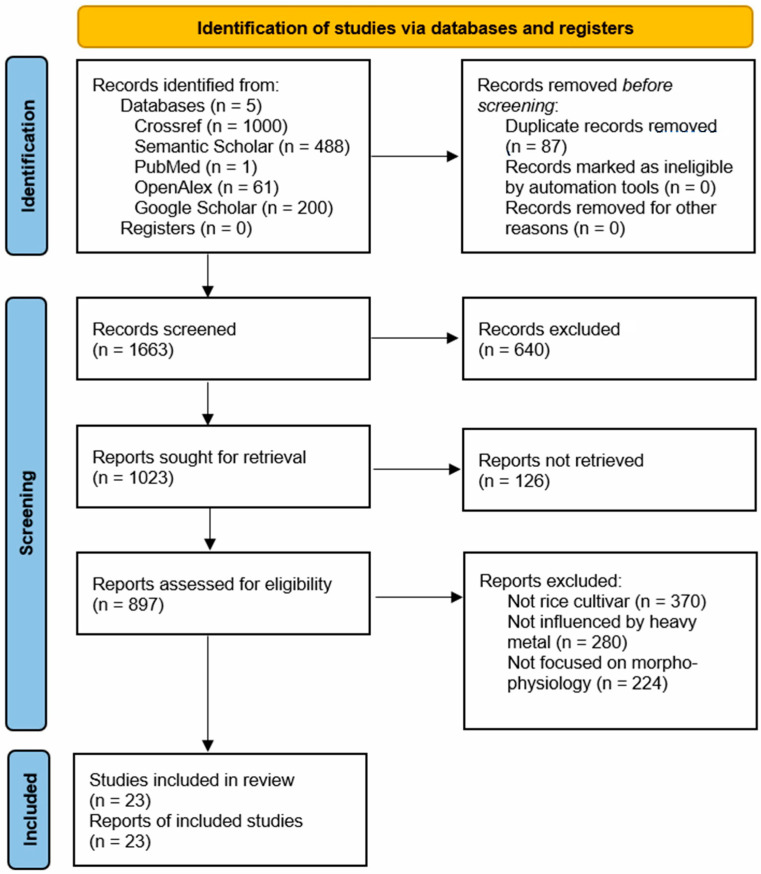
PRISMA flowchart of the search strategy and screening process.

This review followed the PRISMA (Preferred Reporting Items for Systematic Reviews and Meta-Analyses) guidelines for search and screening protocols [21]. Articles were included based on the following criteria: (a) the study must involve any rice cultivar; (b) the focus must be on morpho-physiological analysis; (c) the research must address the effects of heavy metal stress; and (d) the article must be a peer-reviewed research publication within the specified timeframe. Exclusion criteria were also applied, eliminating (a) articles without full text, (b) books, (c) conference proceedings, (d) review articles, and (e) dissertation papers. The initial search yielded 1750 studies related to the effects of heavy metal stress on the morpho-physiology of rice cultivars. After applying the inclusion and exclusion criteria, 23 journal articles qualified for inclusion in the review (Figure 1).

### 2.3. Statistical Analysis

To assess the morphological and physiological changes in rice cultivars due to heavy metal stress, data were analyzed using frequency counts, averages, and percentages. For data standardization, data collected from each article only includes the percentage difference of morphological trait changes and frequency count of the number of rice cultivars that increase or decrease in specific physiological traits. Stacked bar graphs were generated to compare the increasing or decreasing trends in morphological and physiological traits across rice cultivars. For morphological data, the high was set to fifty (50) and the low to negative one hundred fifty (−150), while for physiological data, the high was set to forty (40) and the low to zero (0). All statistical analyses and visualizations were performed using Microsoft Excel.

### 2.4. Use of AI

Grammarly and ChatGPT were used to refine the grammar and improve the clarity of the manuscript and following suggested styles to enhance readability. Turnitin was employed to verify the originality of the manuscript and ensure adherence to plagiarism prevention standards. Importantly, no data were generated by AI tools.

## 3. Results and Discussion

### 3.1. Studies on Heavy Metal Stress on Rice Cultivars

A total of 23 studies investigated the effects of heavy metals on the morpho-physiology of rice cultivars, encompassing 95 unique rice cultivars (Table 1). Nine different heavy metals were examined, with cadmium (Cd) being the most frequently studied (8 studies), followed by lead (Pb) (6 studies). The predominance of cadmium-related research is attributed to its widespread soil contamination through plastic waste, fertilizers, and pesticide residues [22]. Additionally, Cd and Pb are among the most harmful contaminants in agricultural soils due to their toxic effects on plants, including rice, and their broader ecological impacts [23].

Most of the studies used experimental study design to determine the effect of heavy metals on the morpho-physiological traits of rice plants. Specifically, a complete randomized design (8) and a randomized complete block design (6) were used by most of the studies, which indicated the robustness of the studies. Experimental studies are important for drawing specific and reliable conclusions and providing robust associations or causal relationships. Good experimental design is essential and a foundation in plant improvement research [47]. Geographically, the majority of research on the effect of heavy metals on rice plants has been conducted in China (34%) and India (30%), which suggests a higher interest in plant biology researchers in these countries on the topic. This focus may be attributed to the prevalence of heavy metal contamination in these countries, with arsenic (As) and lead (Pb) concentrations in China often exceeding permissible limits for rice cultivation [48]. These conditions likely drive research on the morpho-physiological effects of heavy metals on rice in these areas, given the critical importance of rice as a staple crop.

### 3.2. Morphological Changes on Rice Cultivars Due to Heavy Metal Stress

**Figure 2:**
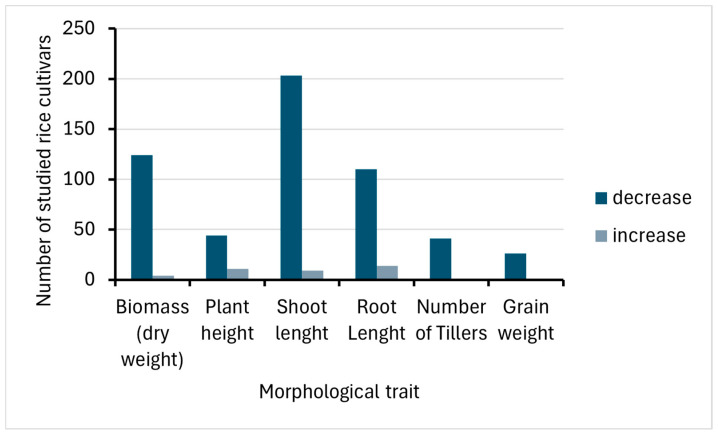
Comparison of morphological trait changes and number of studied rice cultivars under heavy metal stress.

At harvest, various yield components are evaluated to assess the productivity of the rice plant. These components are commonly attributed to the morphological traits of rice, including biomass (dry weight basis), plant height, shoot length, root length, number of tillers, and grain yield [27]. Heavy metals significantly affect various morphological traits of rice. The results of this review indicate a general decline in all traits under heavy metal stress (Figure 2). An in-depth analysis of the concentration and type of heavy metals revealed distinct trends in morphological changes, expressed as percentage increases or decreases relative to control samples.

**Figure 3:**
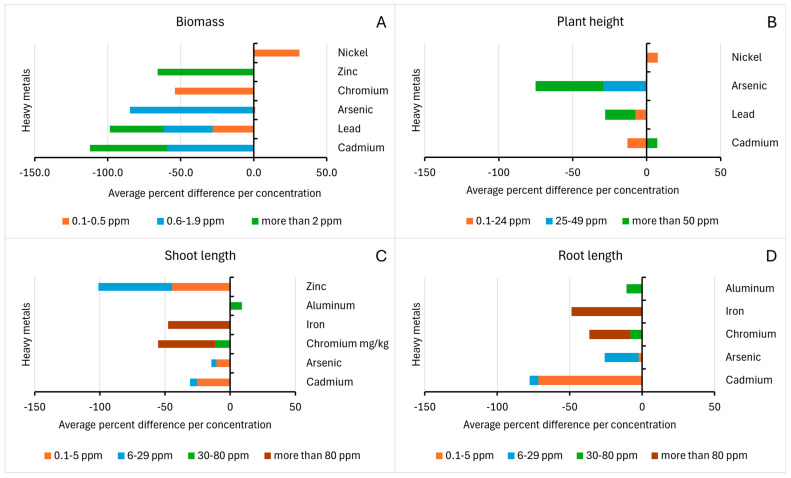
Percentage differences in (**A**) Biomass, (**B**) Plant Height, (**C**) Shoot length, and (**D**) Root length of rice samples based on concentration of heavy metal exposure.

As shown in Figure 3A, the heavy metals cadmium (Cd), lead (Pb), arsenic (As), chromium (Cr), and zinc (Zn) have resulted in a negative percentage difference in biomass as compared to control rice samples which is also consistent for all concentrations: 0.1–0.5 ppm, 0.6–1.9 ppm, and more than 2 ppm. It is also noted from the data of the studies that the increasing concentration of Pb has resulted in a correlated increased percent reduction in the plant’s biomass. A study showed that an increased adverse effect of Pb in the fresh and dry weight of rice is observed at higher concentrations [26]. Although not definitive, the phenomenon can be attributed to the influence of lead in mineral uptake as it restricts the entry of divalent cations such as Ca^2+^, Mg^2+^, Fe^2+^, Zn^2+^, and Mn^2+^, as well as the anion NO_3_^−^, resulting in a nutritional imbalance. In contrast to the above heavy metals, nickel resulted in a positive percentage difference in biomass. This could be attributed to the dual nature of nickel as an essential micronutrient in plants, as it can be promotive at low concentrations but toxic at high concentrations [46]. A low amount of nickel is essential for plant growth as it is a key component of the enzyme urease, which is important for efficient nitrogen assimilation [49], but high amounts can cause biomass reduction, which may be due to less uptake of nutrients, oxidative stress, poor cell wall elasticity, disturbed cell proliferation and inhibition of hydrolytic enzyme activity [50].

Another morphological trait assessed is plant height. As shown in Figure 3B, low concentrations of Ni resulted in increased plant height, similar to the observed effects on biomass, which is attributed to its essential role in nitrogen assimilation. A study included in the review reported an increase in plant height upon high Cd exposure [33]. Between the two rice cultivars used in their study, Super Basmati showed a reduction in plant height, while the change in plant height in JP-5 is considered to have no significant impact. It can be considered that the positive results observed are an outlier, and future studies of the effect of rice cultivars may be employed.

On the other hand, low (0.1–24 ppm) and high concentrations (25–49 ppm) of Cd, Pb, and As have resulted in a negative percentage difference. Cadmium is not essential for plants, and various studies conclude that exposure to Cd results in minimized cell and stunted growth due to reduced nutrient and water uptake, respiration, photosynthesis, and assimilation of nitrogen and carbon [51]. Arsenic, which has no essential role in plants, showed the highest negative percentage difference. The result is corroborated by another study, which noted that As impedes plant growth [52]. The decrease in height can be caused by As phytotoxic effects brought by the escalation of electrolyte leakage, cellular membrane damage, and arresting cell division and expansion [35,52].

For the effects of different heavy metals in the shoot length, Figure 3C shows that exposure to aluminum resulted in a positive percent difference. The shoot height variation depended on the plant genotype, as Al-sensitive cultivar IR64 was shorter compared to Al-tolerant RD35 and AZU [40]. On the other hand, the heavy metal Cd, As, Cr, Fe, and Zn resulted in a negative percent difference, denoting a decrease in shoot length. Zinc, an essential micronutrient and a cofactor in enzymes, exhibited the highest negative percent difference. The decrease is significantly observed in the cultivar Jyothi (JY) and least observed in Varsha (VR), which suggests varying effects based on cultivar tolerance [41]. An average negative percent difference in change in root length is observed for all heavy metals, as shown in Figure 3D. One study, however, reported an increase in root length upon Cd exposure using the cultivar TY816, which is described as a low-Cd accumulation rice cultivar with a highly developed root system developed to cope with Cd stress [38]. Compared to the results in the shoot length, exposure to Al resulted in a decrease in shoot length. For Al-sensitive plant species, a quick inhibition of cell division and cell expansion of root meristem is inhibited, resulting in the observed effect [40]. Moreover, the roots accumulate 30–80% of the total absorbed Al as a strategy to slow down the rate of translocation to other organs, thereby resulting in a more pronounced effect [40]. The negative percent difference in root length compared to shoot length is observed from Cd exposure. This is because when cadmium (Cd) is absorbed through the root cells’ plasma membrane, it damages the root system by inhibiting the formation of lateral roots and making the main root frigid. This is controlled by the electrochemical potential difference between the Cd activity in the cytosol and the root’s apoplast, which drives the uptake of Cd to the other parts of the plant at lower concentrations [51]. Chromium and iron have consistent negative effects on root and shoot length. These effects from various studies conclude that at toxic levels, iron inhibits cell division and the elongation of primary and lateral roots, and chromium negatively affects the uptake of mineral nutrients, reducing the overall morphological growth [53,54].

**Figure 4:**
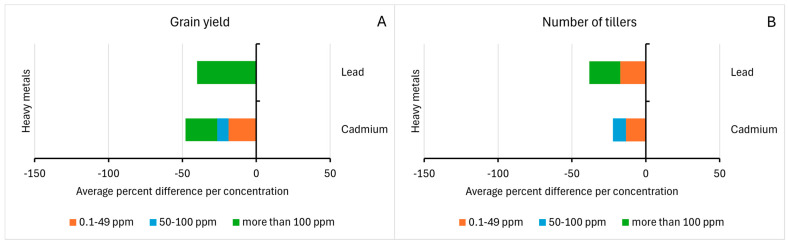
Percentage differences in (**A**) Number of tillers and (**B**) Grain yield of matured rice samples upon Cadmium and Lead exposure.

The last two morphological traits included in the analysis of studies related to the number of tillers and grain yield can give an overview of the effect of heavy metals on the productivity of rice. As shown in Figure 4, both the Cd and Pb in various concentrations have adverse effects on the number of tillers and grain yield of rice.

There is a decrease in the tillering ability of rice with the increasing concentration of both heavy metals. A study noted that the effects of Pb on the cultivar NX-18 are more apparent than other fragrant rice cultivars [25]. The toxic effect of lead is attributed to the disruption of various plant metabolic processes and higher transportation of the metal above-ground parts of the plant. On a similar note, the reduction of grain could be related to the reduction of yield-related attributes under Pb toxicity. A similar study supports the observed trend of significant rice grain yield reduction (59% and 19%) upon high-level Pb exposure (1200 ppm) [55].

Similar to the toxic effects of Pb, an increase in negative percent difference in the number of tillers and grain yield of rice appears to be dose-dependent. The yield loss in terms of the number of tillers and grain yield is due to the reduced nutrient uptake upon Cd exposure, and a decrease in yield parameters reflects the negative impact of Cd on the functions of chlorophyll and its related enzyme activities [42,44]. These findings underscore the strong correlation between heavy metal toxicity, reduced nutrient uptake, morphological alterations, and compromised productivity. Future reviews could benefit from incorporating the economic yield (EY) of the plant, defined as the quantity of grain harvested after the removal of chaff, husks, and other non-grain components, and harvest index (HI), which indicates the efficiency of the plant in producing grains [27]. Results from the literature show an inverse relationship between the plant’s yield (GY, EY, HI) and concentration of heavy metal [45]. Incorporating the analysis of such factors would give insight into the influence of heavy metal accumulation on promoting safe and productive rice cultivation.

### 3.3. Physiological Changes on Rice Cultivars Due to Heavy Metal Stress

**Figure 5:**
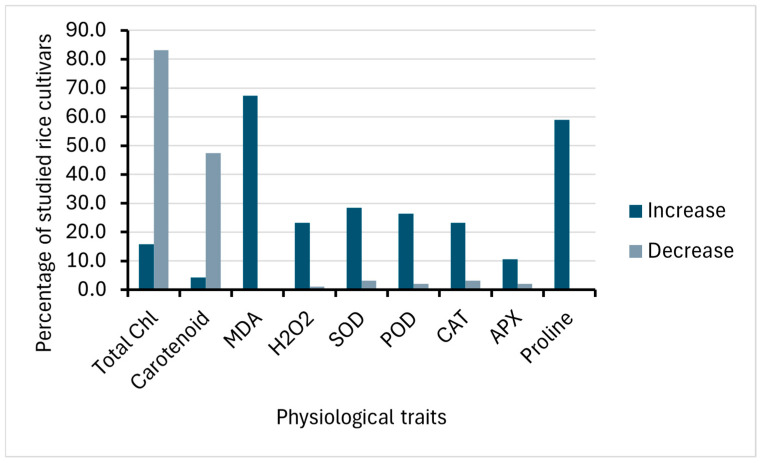
Comparison of physiological traits changes and percentage of studied rice cultivars under heavy metal stress.

Physiological traits analyzed in this review include total chlorophyll content, carotenoid content, malonaldehyde content, hydrogen peroxide content, superoxide dismutase activity, peroxidase activity, catalase activity, ascorbate peroxidase activity, and proline content. Different heavy metals have varied effects on the physiology of rice. Specifically, in terms of total chlorophyll content, most of the studied rice cultivars (83.2%) decreased in total chlorophyll content (Figure 5). The reduction in chlorophyll content was due to the interference of heavy metals to chlorophyll synthase [35,39], an enzyme that catalyzes the final step in chlorophyll biosynthesis. This results in a disturbed photosynthetic activity and supply of energy [37]. However, it is also worth noting that a considerable number of rice cultivars (15.8%) increased in chlorophyll content, which includes PMB/BT/R103, PMB/BT/R178, PMB/BT/R177, PMB/BT/R181, PMB/BT/R007, PMB/BT/R108, PMB/BT/R182, PMB/BT/R185, PMB/BT/R186, PMB/BT/R063, PMB/BT/R184, AZU, IR64, Yuxiangyouzhan, and Meixiangzhan 2 [36,40,46].

**Figure 6:**
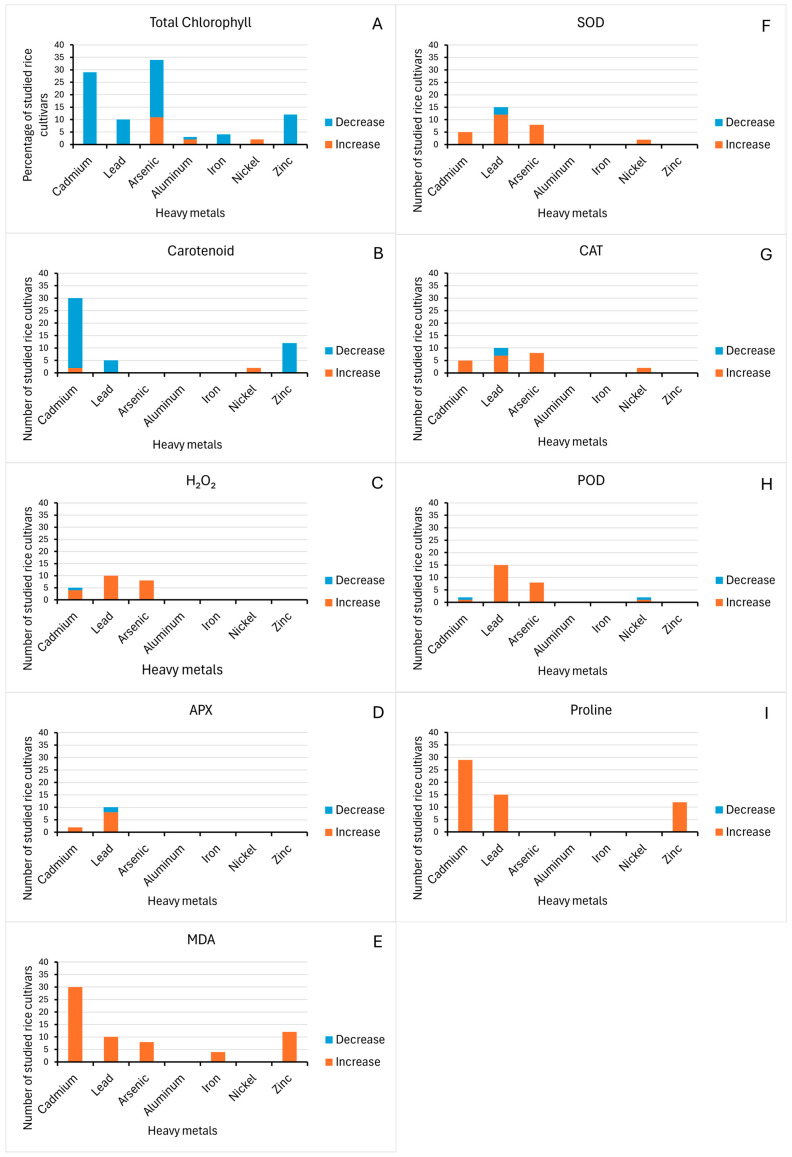
Changes in different physiological traits in response to different heavy metal stress. Physiological traits analyzed were (**A**) total chlorophyll content, (**B**) carotenoid content, (**C**) hydrogen peroxide content, (**D**) ascorbate peroxidase activity, (**E**) malondialdehyde, (**F**) proline content, (**G**) peroxidase activity, (**H**) catalase activity, and (**I**) superoxide dismutase activity.

Maintenance in chlorophyll content could be due to differences in the genotypes, which could indicate a better mechanism of tolerance to heavy metals [36]. Heavy metals reported to have positive effects on the chlorophyll content of some rice cultivars are arsenic, aluminum, and nickel (Figure 6A). In a study on *P. cretica* shoot, arsenic increased Mg content, an important molecule in the synthesis of chlorophyll, elevating chlorophyll in plants, which can be used to counteract arsenic toxicity [56]. The same mechanism could explain the increased chlorophyll content in some studied rice cultivars under arsenic stress. Carotenoid content has a similar response to heavy metal stress compared to chlorophyll; most of the rice cultivars (47.4%) studied decrease in the composition of carotenoid when treated with heavy metals (Figure 5). Reduced carotenoid concentration suggests compromised photosynthetic activity, which might be linked with the inhibition of porphobilinogen synthesis, which would then result in a drop in aminolevulinic acid dehydratase activity, causing chlorosis [29]. However, some rice plants, JP-5, Super Basmati, Yuxiangyouzhan, and Meixiangzhan 2, responded positively, gaining carotenoids under heavy metal stress [33,46]. These rice cultivars gained carotenoids under Cadmium and Nickel treatment (Figure 6B). The improvement of this photosynthetic pigment could enhance photosynthesis in the seedlings, accelerating the rate of dry matter accumulation and carbon metabolism [46].

In addition to effects on photosynthetic pigment, heavy metal stress in plants stimulates the generation of reactive oxygen species (ROS) such as singlet oxygen (^1^O_2_), superoxide radical (*O_2_^−^), hydrogen peroxide (H_2_O_2_) and hydroxyl radicals (*OH) [57]. Reactive oxygen species (ROS) can be generated directly by metals that are active in ROS production via the Haber-Weiss/Fenton reactions, or indirectly through the activation of NADPH oxidases (NOXs), or by obstructing enzymes through the replacement of crucial cations [58]. Increased ROS content can result in oxidative damage, leading to increased lipid peroxidation as indicated by malondialdehyde (MDA) content and the production of an extremely reactive aldehyde called methylglyoxal (MG) [57]. The highly cytotoxic methylglyoxal (MG) is produced during the glycolysis pathway and can induce structural damage to cellular components by forming cross-links that alter proteins, nucleic acids, and carbohydrates [59]. Studies show that under heavy metal stress, such as Cd, plants tend to accumulate a significant amount of MG [29]. MDA production, as a result of the peroxidation of polyunsaturated fatty acids, is a biomarker of the degree of membrane damage and is, hence, a key determinant of oxidative stress [25]. All studied rice cultivars (67.4%) that examined malondialdehyde exhibited an increase in malondialdehyde (MDA) content (Figure 5), a substance that is formed by membrane lipids in response to reactive oxygen species which can be used to assess the extent of damage of heavy metals to the plasma membrane, and studies showed all heavy metals promoted MDA production (Figure 6E). Elevated production of MDA suggests cadmium susceptibility due to extensive membrane damage [29]. The activation of this antioxidant indicator has also been reported in different studies in rice, wheat, and maize [37,60,61].

Most of the studied rice cultivars have enhanced production of H_2_O_2_ in response to different heavy metals (Figure 5). The increase in H_2_O_2_ with increasing concentrations of heavy metals like cadmium and arsenic suggests that the heavy metals promote oxidative stress in rice plants [24,33]. Specifically, Pb stress frequently causes the synthesis of reactive oxygen species (ROS) and free radicals, which bring ultrastructural and functional modifications in cell nuclei, DNA, lipids, and proteins [24]. Not all the studied rice cultivars have increased production of H_2_O_2_. Super Basmati was found to have reduced H_2_O_2_ content (Figure 6C), thereby avoiding oxidative stress in an environment with high Cd concentration [33]. This suggests that Super Basmati could tolerate Cd stress, specifically oxidative stress, compared to other rice varieties, which respond to heavy metals by enhancing the production of H_2_O_2_.

Plants have a dual antioxidant defense system that scavenges ROS production in plants. The first comprises non-enzymatic antioxidants such as ascorbate (AsA), glutathione (GSH), and phenolic compounds, while the latter comprises enzymatic antioxidants such as peroxidase (POD), superoxide dismutase (SOD), catalase (CAT), and ascorbate peroxidase (APX) [57]. In the scavenging of reactive oxygen species (ROS), the plant employs both enzymatic and non-enzymatic antioxidant mechanisms. In the context of mitigating cadmium (Cd) toxicity, glutathione (GSH) plays a crucial role in the ascorbate– glutathione (AsA-GSH) cycle, where ascorbate peroxidase (APX) detoxifies hydrogen peroxide (H_2_O_2_) in coordination with monodehydroascorbate reductase (MDHAR), dehydroascorbate reductase (DHAR), and glutathione reductase (GR). In addition, GSH is involved in the elimination of MG via the glyoxalase (GLY) pathway [62]. Apart from ROS, it is noted that sulfur-rich ligands such as GSH and phytochelatins are involved in mitigating toxic levels of heavy metals by directly binding and sequestrating the metal into the vacuoles. A study involving Cd toxicity showed a dose-dependent relationship with phytochelatin in response to increasing stress [59].

Superoxide dismutase (SOD) is a metallo-enzyme that helps detoxify reactive oxygen species (ROS), which protects cellular components from being oxidized [26,63]. A majority of studied rice cultivars have increased SOD activity due to heavy metal stress (Figure 5). When SOD activity increases, reactive oxygen species, particularly the superoxide radical, can be detoxified by the plant more easily [37]. As biotic and abiotic stressors are present, SOD activity usually rises in proportion to the level of stress. Increased SOD activity means that the plant is strengthening its antioxidant defenses against oxidative damage in response to heavy metal stress. This lessens the negative consequences of oxidative stress by preserving cellular integrity and function. Some of the rice cultivars were observed to have decreased SOD activity, such as XYXZ, NX-18, and Meixiangzhan-2 (Figure 6I). This implies that they are deficient in detoxifying ROS, especially the superoxide radical (O^2−^). This decline could be a sign that the plant’s antioxidant defenses are being overloaded by heavy metal stress or that the synthesis or functionality of SOD enzymes is being hampered.

The activity of peroxidase could indicate rice’s oxidative defense against oxidative damage brought on by heavy metals. In this review, most of the studied rice cultivars increased in POD activity (Figure 5). This increased POD activity shows that the rice seedlings’ H_2_O_2_ levels have decreased, demonstrating a successful alleviation of the oxidative damage brought on by different heavy metal exposures. This was observed in different studies showing the mechanism of plants to protect themselves from oxidative damage as it maintains cellular homeostasis, strengthens the cell wall, and detoxifies harmful substances under heavy metal stress [37,64,65]. However, based on the review, two rice cultivars, JP-5 and Meixiangzhan 2, were observed to have decreased POD activity, which was observed in cadmium and nickel studies (Figure 6G). The decreased POD activity indicates that the adaptive response of rice plants to reduce the adverse effects of heavy metal toxicity is compromised.

One of the most significant antioxidant enzymes that can reduce or even completely halt the oxidation of biomolecules is catalase, which, as a result, can assist plants in tolerating a particular level of hazardous metals in soil [66]. This review also reveals that most of the studied rice cultivars have increased catalase (CAT) activity in response to several heavy metals (Figure 5). Elevated catalase activity means that the rice plant is trying to enhance its antioxidant defense system to counteract the harmful effects of the heavy metals by converting harmful ROS into water and oxygen [26]. Although this is an expected response of a plant to counteract heavy metal stress, there are still some rice varieties that are negatively affected, such as Meixiangzhan-2, Xiangyaxiangzhan, and Basmati-385, which was observed in Pb treatment (Figure 6H). This indicates that the capacity of these plants to counter oxidative stress is inhibited. This research further demonstrates that plant genotype, the degree of stress to which plants are exposed, and metal content all influence whether enzymatic activity rises or decreases [67].

Ascorbate peroxidase activity (APX) was observed to increase in most rice cultivars that were studied under different heavy metal stress (Figure 5). Another antioxidant enzyme found in plants is APX, which is essential for reducing oxidative stress brought on by heavy metals in particular. It is a vital component of the ascorbate–glutathione cycle, catabolizing ascorbic acid to dehydroascorbate and aiding in the chloroplast’s conversion of H_2_O_2_ to H_2_O [24]. APX increase means that the plants are fighting oxidative stress in plants brought on by heavy metal toxicity [68]. On the other hand, Meixiangzhan-2 and NX18 were observed to have decreased APX activity. The decrease in APX activity was also observed in the roots of Cd-treated plants [69].

Osmolytes accumulating in the cytosol are another defense mechanism in plants in response to stress conditions [18]. These inert solutes include sugars, polyamines, secondary metabolites, and amino acids. Osmolytes help preserve membrane integrity, regulate cellular osmotic pressure, detoxify reactive oxygen species, and sustain cell turgor by mitigating dehydration and damage [70]. The amino acid proline functions as a remarkable osmolyte, having key roles in stress regulation, such as maintaining cell osmotic pressure and regulation on ion transport and detoxification of heavy metals [27,35]. Proline content increased in all studied rice cultivars due to different heavy metal stress (Figure 5 and Figure 6F). Proline accumulation has been observed in a variety of plants under heavy metal stress [71]. Increased proline accumulation was notably observed in Tunnae and Mashrab, which are Pb-tolerant rice cultivars, highlighting the important role of proline in the adaptive mechanism for Pb stress tolerance in rice [36]. In addition to its osmoprotective and ROS-quenching properties, proline also functions as a heavy metal chelator, which alleviates heavy metal stress. Specifically, proline causes the formation of phytochelatins that chelate with heavy metals such as Cd, reducing their toxicity [71].

## 4. Conclusions

This review aims to provide a comprehensive summary of the rice cultivars investigated, the heavy metals studied, the experimental designs employed, and the geographic locations of sampling and study sites while analyzing the morphological and physiological changes in rice cultivars subjected to heavy metal stress. The analysis identified 23 experimental studies examining 95 different rice cultivars in response to nine heavy metals. Notably, the majority of these studies were conducted in China and India, reflecting the significant interest of plant biology researchers in these regions in this topic.

The findings demonstrate that exposure to heavy metals generally leads to a decline in all morphological traits. Heavy metals such as cadmium (Cd), lead (Pb), arsenic (As), chromium (Cr), and zinc (Zn) adversely affect a plant’s ability to absorb essential minerals, maintain nutrient balance, preserve cellular membrane integrity, and undergo cell division, resulting in reduced biomass and plant height. However, nickel (Ni) exhibited a unique effect, showing a positive percentage change in biomass, likely due to its dual role as both a micronutrient essential for plant growth and a stressor at higher concentrations. Deviations from the overall trend of decreased shoot and root length, tiller number, and grain yield were observed in specific cultivars. For instance, cultivars RD35 and AZU, known for their aluminum (Al) tolerance, exhibited longer shoots under Al exposure, while cultivar TY816, characterized by minimal Cd accumulation, demonstrated longer roots under chromium (Cr) stress. Additionally, the aromatic rice cultivar NX-18 exhibited the greatest reduction in tiller number compared to other aromatic rice varieties, highlighting variability linked to genotype. A dose-dependent relationship was also observed between reductions in tillering ability and grain yield with increasing concentrations of Pb and Cd.

In terms of physiological responses, the analysis revealed a general trend of decreased total chlorophyll and carotenoid content, accompanied by increases in markers of oxidative stress, such as malondialdehyde (MDA) and hydrogen peroxide (H₂O₂), as well as antioxidant enzyme activities, including superoxide dismutase (SOD), peroxidase (POD), catalase (CAT), ascorbate peroxidase (APX), and proline content. However, exceptions to this trend were noted, with seven rice cultivars showing increased total chlorophyll content and four cultivars exhibiting increased carotenoid levels, suggesting a degree of tolerance to heavy metal stress in these genotypes. Conversely, reductions in SOD, POD, CAT, and APX activities were observed in 10 cultivars, indicating a compromised adaptive response to oxidative stress caused by heavy metal accumulation.

In conclusion, this review underscores the significant morphological and physiological impacts of heavy metal stress on rice plants, with responses varying by genotype, metal type, and stress intensity. While most cultivars exhibit declines in growth, yield, and chlorophyll content, exceptions such as tolerant cultivars highlight the potential for breeding stress-resilient varieties. The dose-dependent effects of heavy metals further emphasize the urgency of managing soil contamination to safeguard agricultural productivity and food security. These findings provide a foundation for future research and strategies to mitigate heavy metal stress in rice cultivation.

### 4.1. Economic and Agronomic Implications

Based on the results, heavy metals such as cadmium (Cd), lead (Pb), arsenic (As), and chromium (Cr) negatively impact important morphological traits that have a direct bearing on productivity, such as biomass, plant height, and grain yield. Physiologically, plant development and reproductive success are disrupted by these metals as they cause oxidative stress, interfere with nutrient uptake, and impede photosynthesis. Economically, rice contaminated with heavy metals not only reduces yield but also makes harvested grain unsafe for human consumption, resulting in market losses and health risks. This puts further financial strain on farmers as it necessitates increased costs for remediation strategies such as soil amendments, cultivar selection, and agronomic interventions. Addressing heavy metal contamination in rice fields is essential for ensuring food security, economic sustainability, and public health, especially in areas where rice is a staple crop and the main source of income.

### 4.2. Limitations

The study has a few methodological limitations that affect the applicability and relevance of its findings. First, as the included studies differ in their experimental designs, methods, and reporting standards, the use of secondary data from these published literature may induce biases. Differences in sample sizes, measuring methods, and environmental conditions may result in inconsistent data, which can affect the generalizability of the results. Second, despite using every available resource, the researchers obtained a small number of included studies for this review, limiting the applicability of the findings. Lastly, the use of a simple statistical analysis, while easily accessible and user friendly, limits the complexity and rigor of the analyses that were performed.

## Figures and Tables

**Table 1:** Studies on heavy metal stress on rice cultivars indicate the study design and sampling location.

Heavy Metals	Rice Cultivars	Study Design	Sampling Location and Study Site	Reference
Lead (Pb)	Guixiangzhan (GXZ) and Nongxiang-18 (NX-18)	Experimental	Guangzhou, China	[24]
Lead (Pb)	Meixiangzhan-2, Xiangyaxiangzhan, and Basmati-385	Experimental	Guangzhou, China	[25]
Lead (Pb)	Meixiangzhan (MXZ-2), Xiangyaxiangzhan (XYXZ), Guixiangzhan (GXZ), Basmati-385 (B-385), and Nongxiang-18 (NX-18)	Experimental	Guangzhou, China	[26]
Copper (Cu), Zinc (Zn) and Lead (Pb)	Not specified	Not specified	Perlis, Malaysia	[27]
Chromium (Cr)	Not specified	Experimental	Hangzhou, China	[28]
Cadmium (Cd)	Maharaj, Pratiksha and Khitish	Experimental	West Bengal, India	[29]
Lead (Pb) and Chromium (Cr)	Yongyou 538, Chunyou 84, Jiahe 218, and Xiushu 134	Experimental	Zhejiang Province, China	[30]
Chromium (Cr)	Pratikshya (ORS 201-5), Bina Dhan 11(Ciherang Sub-1), and Kalachampa (FV-152)	Experimental	Bhubaneswar, India	[31]
Cadmium (Cd)	Giza 177–179, Giza 181–182, Sakha 101–106, Egyptian Yasmin	Experimental	Kafr EL-Sheikh, Egypt	[32]
Cadmium (Cd)	Indica and Japonica	Experimental	Islamabad, Pakistan	[33]
Arsenic (As)	BRRI-33, BRRI-39, BRRI-44 and BRRI-51	Experimental	Rajshahi, Bangladesh	[34]
Arsenic (As)	Nipponbare, Indica rice 9331, Guiyin 206, Minghui 63, Zhenong 34, Zhenong 41, Zhenong 37, and Shanghai 1	Experimental	Hangzhou, China	[35]
Arsenic (As)	Birohi dhan, Tora Sali, Doom Sali, Biyoi Sali Bora, Ijong, Kethakeni Boni, Lota Sali, Bora, Johaboni, Jaya, Boro, Baismuthi, Moinagiri, Ijong, Balam, Chanmoni, Masuri, Ranjit, Sok Bonglong, Maiju Hankar, Sapna, Monasali	Experimental	Assam, India	[36]
Lead (Pb)	Yasmen, Amber Barka, Tunnae, Ilmi, and Mashkab	Experimental	Daegu, Korea	[37]
Cadmium (Cd)	Jianyou841 and Teyou 816	Experimental	Guangzhou, China	[38]
Cadmium (Cd)	IR64, Satabdi, Bandana, Palman, Swarna, Khanika, Kariagora, Badshahbhog, Gobindobhog, Tulsibhog, Pusa Basmati, Tulaipanji, and Rabhunipagol	Experimental	West Bengal, India	[39]
Aluminum (Al)	IR64, Azucena (AZU), and RD35	Experimental	Nakhon Pathom, Thailand	[40]
Zinc (Zn) and	Aathira (AR), Aiswarya (AS), Annapoornna (AP), Jyothi (JY), Kanchana (KN), Karuna (KR), Mangalamahsuri (MM), Mattatriveni (MT), Neeraja (NR), Swarnaprabha (SP), Swetha (SW), and Varsha (VR)	Experimental	Kerala, India	[41]
Cadmium (Cd)	BRRI dhan48	Experimental	Dhaka, Bangladesh	[42]
Iron (Fe)	IR64, Inpara 5, Inpara 2, and Pokkali	Experimental	Bogor, Indonesia	[43]
Cadmium (Cd)	Pusa Basmati 1121	Experimental	Punjab, India	[44]
Cadmium (Cd)	Pusa Basmati 1121	Experimental	Punjab, India	[45]
Nickel (Ni)	Meixiangzhan 2 and Yuxiangyouzhan	Experimental	Guangzhou, China	[46]
